# Opening Pandora’s Box: Peeking inside Psychology’s data sharing practices, and seven recommendations for change

**DOI:** 10.3758/s13428-020-01486-1

**Published:** 2020-11-11

**Authors:** John N. Towse, David A Ellis, Andrea S Towse

**Affiliations:** 1 Department of Psychology, Lancaster University, Lancaster, LA1 4YF UK; 2 School of Management, University of Bath, Bath, BA2 7AY UK

**Keywords:** Open science, Public data sharing, Transparency, Reproducibility, Meta-research

## Abstract

Open data-sharing is a valuable practice that ought to enhance the impact, reach, and transparency of a research project. While widely advocated by many researchers and mandated by some journals and funding agencies, little is known about detailed practices across psychological science. In a pre-registered study, we show that overall, few research papers directly link to available data in many, though not all, journals. Most importantly, even where open data can be identified, the majority of these lacked completeness and reusability—conclusions that closely mirror those reported outside of Psychology. Exploring the reasons behind these findings, we offer seven specific recommendations for engineering and incentivizing improved practices, so that the potential of open data can be better realized across psychology and social science more generally.

**Supplementary Information:**

The online version of this article (10.3758/s13428-020-01486-1) contains supplementary material, which is available to authorized users.

## Introduction

Data archiving and public data-sharing for published research can make an important and positive contribution towards a more open-research culture—increasing research credibility and enhancing research integrity. We use *public data-sharing* as a term synonymous with *open data* (see Martone, Garcia-Castro, & VandenBos, 2018). We recognise that the term data sharing has previously referred to restricted data release or for example, peer-peer exchange (see Houtkoop et al., 2018). At a minimum, such public data-sharing allow results to be checked and validated by others. Yet benefits extend much further—open data may also be used to facilitate data aggregation (e.g., for meta-analysis), permit creative re-analysis (e.g., combining or using data in new ways) or assist with later scientific developments (e.g., new statistical or methodological techniques can be retro-fitted to existing findings). Providing open data also responds to the political manifesto that, so far as is possible, publicly funded work should be publicly accessible.

The broad recognition of the value of open data is happening in parallel with the adoption of scalable technical infrastructures such as Digital Object Identifier (DOI) standards (Davidson & Douglas, 1998) and data management processes (Sturkis & Read, 2015) that facilitate implementation of public data-sharing practices. Online academic journals can curate far more than printed words in a research output adding value to their collections. Data storage options are also increasing, some of which are based in institutions, some embrace the bespoke service needs of particular disciplines or funding agencies, whilst others such as osf.io are available to any researchers. Although systems for locating, maintaining and visualizing data have become more sophisticated, these new resources also bring challenges; for the researcher, in the time required to prepare materials; for the user, in the navigation, organization and understanding of the archived files and systems (Ellis & Merdian, 2015).

A recent report suggests that public trust in scientists is heightened when data are openly available (Pew Research Center, 2019). However, this assumes that the datasets are functional, for example by conforming to FAIR principles (Wilkinson et al., 2016). These focus on how data should be Findable (e.g., have a persistent identifier and descriptive meta-data), Accessible (ideally available without authentication requirements or data sharing restrictions, with metadata to clarify any conditions or accessibility issues even if raw data are not available), Interoperable (use common standards for description), and Reusable (appropriately licensed and meaningful). Nonetheless, despite considerable interest in data sharing as an *ideal* (Munafò et al., 2017) the extent to which public data sharing occurs across Psychology is unclear, and more critically the extent to which open datasets are useful is even less well understood. In the current paper, therefore, we systematically evaluate the functionality of open data across Psychology. In doing so, we deliberately draw on influential work by Roche, Kruuk, Lanfear & Binning (2015; hereafter RKLB), who investigated open datasets in Ecology & Evolution, so as to permit comparisons across science and draw on their methods and insights for considering data quality.

RKLB sampled open datasets accompanying papers in the field of Ecology & Evolution published in 2012 and 2013, and surveyed the quality of these datasets in terms of their completeness (addressing whether *all* the data and data descriptors supporting a study’s findings are publicly available) and reusability (asking how readily the data can be accessed and understood by third parties). These scores explicitly incorporate the FAIR principles, but also go beyond them—for example by examining in detail how well data descriptions allow researchers to map data points to experimental designs and results in the source research paper. They also considered licensing or availability of file formats, not just licensing of the data themselves. They observed a striking variability across sampled datasets, which ranged from exemplary to indecipherable. Moreover, the overall profile was alarming—the *majority* of the datasets were incomplete, and the majority had limited re-reusability. In other words, many datasets (and thus the science base) were not FAIR, they were instead limited by researcher practice. This had the effect of rendering large swathes of data “reuseless” (Mons et al., 2017). Developing their methodology incrementally, we sought to establish if their unnerving portrait is also true for Psychology.

We chose to make some specific alterations to the original RKLB methodology. RKLB drew on data held only in a single repository (Dryad). We were not constrained as to how the data could be made public because our starting point was a systematic sampling search of journal papers. First, this allows us to describe the historical prevalence of open data provision in psychological journals. Second, since datasets might be archived in different ways, we could make a more representative assessment of the “Findable” and “Accessible” elements within FAIR. Federer et al. (2018) highlight this issue in discussing mandated data availability statements for one mega-journal, since they concluded that the majority of statements (for papers published 2014–2016) were not Findable and Accessible (especially where they were claimed available on request).

Hardwicke et al. (2018), in work that emerged as a preprint at the time of our study preregistration, analysed both the frequency of data sharing and also characteristics of the deposited data in a single psychological journal, *Cognition*. One focus was the change to the data policy of that specific journal (and so they compared data sharing before and after a mandatory open data policy came into effect) using their own metrics to assess data sharing practices. Our current work is complementary to Hardwicke et al. and also uses the same publication window (i.e., 2014–2017). We apply a much broader approach by incorporating multiple journals across the discipline (and accordingly fewer papers from each outlet). By measuring data functionality in the same way as RKLB, we are able to compare datasets in Psychology with those in Ecology & Evolution—this alignment is crucial to appreciate the specificity or generality of dataset functionality issues across disciplines.

Our pre-registration set out the following broad research question: do researchers publishing in Psychology make fully functional data deposits? To address this question, the study primarily planned to establish:

- the completeness of data (as defined by RKLB)
- the reusability of data (as defined by RKLB)

in so doing, the preregistration additionally set out a protocol to establish, as a by-product of addressing (a) and (b)

c) the prevalence of open data provision across journal articles

To address these three questions, we examined 15 Journals at each of two separate time periods. We should also note that, as we describe in more detail later, our assessments of data quality go beyond just those developed by RKLB. So, the first two questions (a & b) provide a disciplinary comparison or starting point and framing for a broader examination of data functionality.

### Methodology

As recommended by Simmons, Nelson, and Simonsohn (2012), *we report how we determined our sample size, all data exclusions, all manipulations, and all measures in the study*.

### Journal selection

Across two study pre-registrations (see below), we identified 15 psychology journals. Journals were chosen in order to generate variability in:

- psychological content (i.e., drawing on different areas within the discipline)
- connections with the academic community (we ensured a mixture of society-owned and unaffiliated journals).
- publication formats (i.e., hybrid vs exclusively *Open Access (OA) journals*).
- Involvement of different publishing companies.
- Impact factors (accordingly, we only considered journals with impact factors, and thus some publication longevity).

Some journals had more explicit data sharing policy than others, but this was not considered formally in journal selection. As authors, we drew on our collective experience in social psychology, cognitive science (including developmental psychology) and applied research to select the journal set.

### Data acquisition

**Fig. 1:**
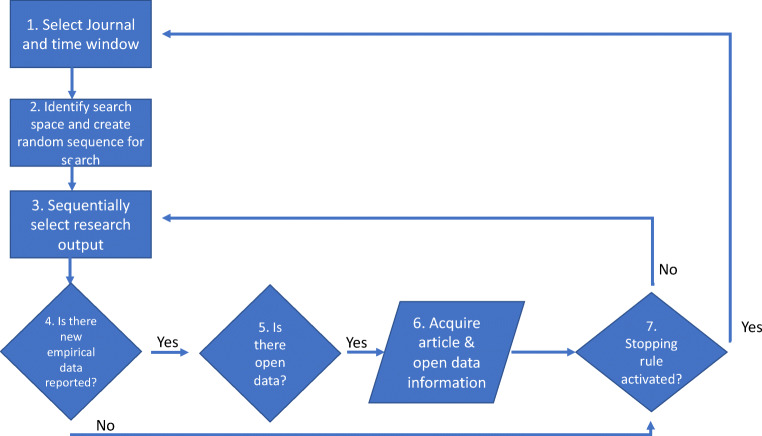
Process flowchart to describe the data acquisition process. Step 1 involved 15 journals each with two time windows (i.e., 30 cycles of data acquisition). Step 4 involved an examination of 2243 research outputs, of which 1900 were considered in step 5, and which identified 71 datasets in step 6

A schematic overview of the data acquisition process is shown in Fig. 1. This was designed to acquire our target data corpus of 120 datasets (similar but somewhat larger than that reported in RKLB).

For each selected journal, we derived a comprehensive catalogue of all published articles within the pre-defined publication window. We searched these in a random order (provided by random.org) to avoid biases from any sequential or chronological journal differences.

For each article, we used the DOI to find the source online and we then manually searched for an identifiable dataset. We looked *throughout* the paper, including footnotes, author notes and supplementary information presented on the landing page for the paper where relevant, but we focused especially the methods and the results section—where there might be specific links to material and data. We also recognized that the data may be in the paper itself, in the form of a table or appendix.

For each paper, the search could result in one of the following outcomes:

- identification of open data
- categorization as a research output with no underlying data (e.g., a theoretical commentary, technical descriptions, editorials or corrigenda etc.)
- an empirical paper with no data explicitly linked at the time of publication. We included in this category papers involving secondary data analysis only where the extracted data, or further-processed data, could have been used to create a fresh dataset. We excluded papers where the text described an example data point or text extract, in other words any data were for explanatory purposes only.

We employed a search stopping rule once we found four datasets in the selected publication window (four articles in each of two different time windows for 15 journals). For some journals, we examined all articles in the specified time period and did not find four datasets reducing the size of the open dataset corpus (e.g., for one journal we only found two datasets in 88 papers), which inevitably lead to a second stopping rule. There were six searches (out of 15 journals x 2 time periods) that produced exhaustive examination of all papers without identifying four datasets. After pre-registration, we agreed on the necessity of an additional stopping rule—we curtailed our search after examining 100 papers per journal per time point. We did so after establishing that some journals had both very large publication volumes (close to 1000 papers) and very low adoption rates.

All three authors examined journals for datasets, with AT performing most of the searches. We resolved emergent issues by mutual discussion (e.g., the need for the third stopping rule, thresholds for what constitutes open dataset vs. an illustrative data point, etc.) and a proportion of articles and datasets were examined by more than one author. Search statistics are detailed in our data deposit.

Our initial study pre-registration plan, identified ten journals with dataset sampling proposed across three separate time periods (2012/2013, 2014/2015, and 2016/2017). This incorporated the publication window examined by RKLB for Ecology & Evolution (2012/2013) and two subsequent time windows. However, information mostly from 2014/15 searches alongside a single journal search from 2012/13 made clear that we would not find enough datasets from 2012/3. Consequently, we submitted an amended pre-registration prior to any data analysis, in which we dropped the earliest publication period and added additional journals (*n* = 5) to compensate for the reduction in the dataset corpus. The second preregistration references the first and explains the adapted plan.

Details of the open dataset search process, and the analysis of the data themselves, is documented in our accompanying data deposit (https://osf.io/2fpgc). Our approach to describing and reporting data quality follows RKLB, and we too have masked the dataset so the papers sampled cannot be directly identified.

### Scoring data quality

We examined each dataset and derived measures of completeness and reusability, implementing the ordinal scale described by RKLB (their protocol is reproduced in the supplementary materials section and can be found here). Accordingly, “5” is exemplary, “4” is good, “3” represents small omission / average, “2” represents large omission / poor while “1” is poor / very poor respectively. For clarity we also recorded “0” in cases of no data (see below). For completeness, these scores reflect the ability to understand the dataset independent of the paper, and the ability, in principle, to reproduce all or some of the analyses from the paper. Where some data or explanations are missing, the score is lower. For reusability, the scores indicate whether the data are machine readable, whether they rely on proprietary software, and whether metadata are informative for understanding the dataset.

We also measured other features of each dataset, for example the number of files, whether units of measurement were specified, the analysis software used (where identifiable) and whether analysis code was provided. Like RKLB, we annotated each dataset evaluation.

**Fig. 2:**
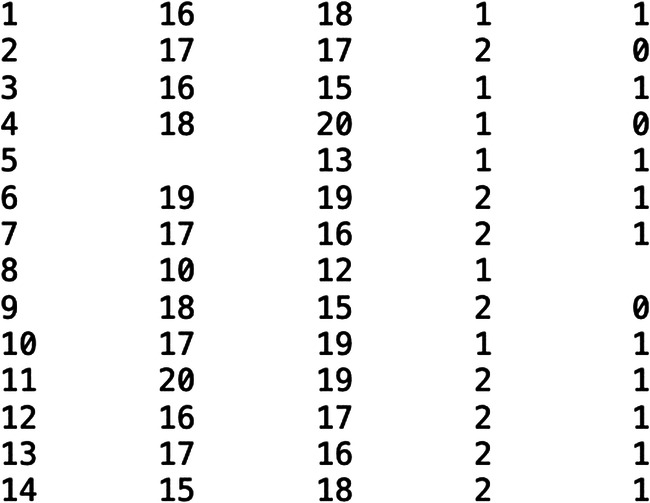
Fictitious archived dataset file

Consider the following fictitious dataset in Fig. 2, from a research study describing a stereotypical 2 x 2 experiment analysed for 12 participants (nb., although artificial, what follows is grounded in some of our analytic experiences). The study investigated the ability to distinguish previously presented and unfamiliar stimuli under two conditions (low stress and high stress) and as a function of whether the participant was a psychology student or an English student. Imagine this is all you have available to work from.

Some issues become immediately apparent. What do the columns represent? Without explanation, there is varying degrees of ambiguity. The first column would clearly make sense as a participant number, but that nonetheless requires an inference (e.g., could the dataset be ordered /sorted by this variable instead?) and note that the number of participants this implies (14) does not match the stated analysis in the paper (12). The second and third column could represent recall performance in the two (low and high) stress conditions. However, which is which? Indeed, this could instead represent first test and second test performance, with a separate column indicating the mapping between stress condition and test order. The study recruited students from two degree courses, but which column, if any, describes this, and which cell value represents which category? Separately, there are empty cells in this dataset, in the 2nd and 5th column, but are these missing or deleted data, or a transcription / archiving error, etc.?

On the other hand, if there was an accompanying codebook / data description / readme file, then several /all of the above problems might be resolved. Moreover, the codebook potentially describes in richer detail what the columns represent (e.g., for the putative recall data in column 2, that this involved a total score of correct recalls and correct rejections of lure stimuli). Furthermore, in some cases, alongside these data used for a 2 x 2 analysis on recall totals, there might also be files for each individual participant, detailing exactly which stimuli were presented at encoding and at test.

In terms of accessibility and reusability, the file format used for these data would be relevant. A comma separated file is readable as text and by non-commercial software. On the other hand, if the data were held as an image-based pdf, it could not be read by most analysis software, or if an SPSS (.sav) file, it is essentially encrypted without the user having access to a current, commercial, SPSS license.

#### Training in dataset assessment

In personal communication with Roche (July 2018), we discussed our project aims and obtained identification information about the datasets in the original study. This allowed the primary rater (AT) to check and corroborate scoring for a sample of four RKLB datasets. In other words, we trained ourselves on scoring using both published RKLB materials that explained their scoring rules (see supplementary materials), but also validated with the some original RKLB data. After the current corpus had been evaluated, secondary coders (JT & DE) blindly sampled seven datasets (10%) for both completeness and reusability. 28/42 pairwise ratings matched exactly, and 35/42 pairwise ratings differed by 0 or 1 (e.g., one rater was more or less generous in the categorization of a “minor” data omission). Discursive internal review of these scores confirmed the absence of systematic biases in ratings (i.e. direction of differences was variable) and emphasized confidence in the primary scores. This rater agreement exercise is documented in the data deposit.

## Results and discussion

### The prevalence of open data in Psychology

Our primary focus involves the quality of open data, but we begin by documenting its prevalence as it sets a context for the work that follows, and this was undertaken first.

Our journal search led us to examine 2243 independent output contributions. Of these, 1900 papers were eligible empirical papers (*prima facie*, authors could have made a data deposit) from which we acquired a corpus of 71 datasets. The adoption rate of open research data across all journals and time periods is approximately 4%. Prevalence increased between the two time periods (26 datasets/1065 searches = 2.44%, vs. 45/835 = 5.39%), with a significant and large effect comparing adoption rates for each journal across time point, F(1,14) = 5.44, *p* = .035, η^2^ = .280. This is a low base rate of public data sharing, but just as striking is the variability in adoption rate across journals.

**Fig. 3:**
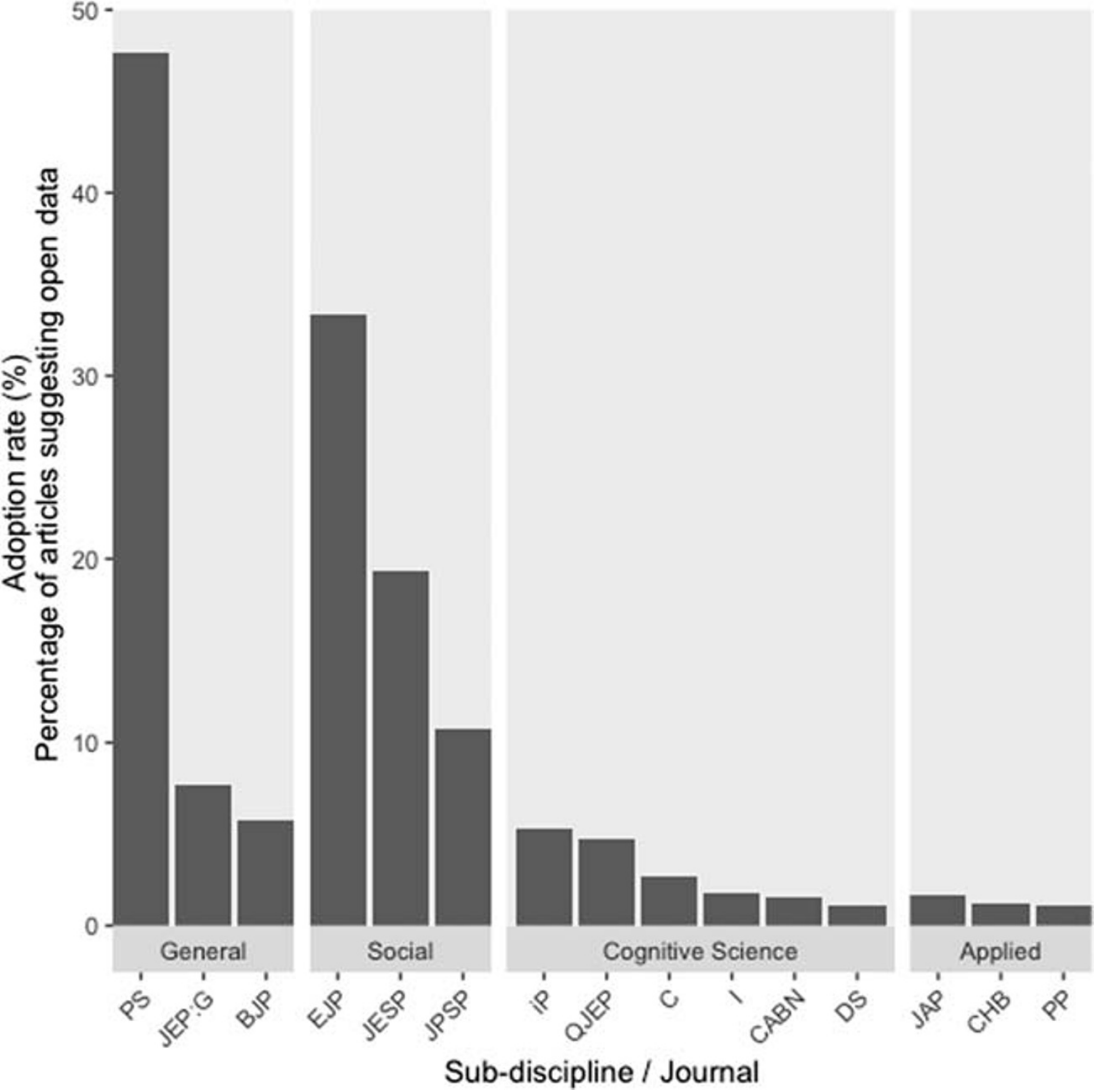
Open dataset adoption rate for each journal collated by journal area. Journals (ordered by open data prevalence for each group): (a) General – (PS) Psychological Science; (JEP:G) Journal of Experimental Psychology: General; (BJP) British Journal of Psychology (b) Social – (EJP) European Journal of Personality; (JESP) Journal of Experimental Social Psychology; (JPSP) Journal of Personality and Social Psychology; (c) Cognitive science – (iP) i-Perception; (QJEP) Quarterly Journal of Experimental Psychology; (C) Cortex; (I) Infancy; (CABN) Cognitive, Affective & Behavioral Neuroscience; (DS) Developmental Science; (d) Applied – (JAP) Journal of Abnormal Psychology; (CHB) Computers in Human Behavior; (PP) Personnel Psychology

We had deliberately chosen the 15 journals to reflect a variety of outlets and cover different areas of Psychology. We averaged data from each time period and chose *post-hoc*^1^ to organize them into outlets covering “social” psychology, “applied” psychology, “cognitive science” (including neuroscience and developmental psychology) and “general” (journals in which the sub-discipline is not specified, and all the above areas would be appropriate). Figure 3 illustrates how social and general journals that we sampled include a higher proportion of open datasets (nb., the accompanying online data deposit illustrates adoption rate for each journal at each publication time point, as well as separately providing an interactive sunburst plot of these data here).

It is worth noting that journal sub-discipline was not systematically manipulated. We argue that these sub-disciplines are reasonable and meaningful for the community, but they are only one lens through which to view the corpus. Although the results are intriguing, they are not conclusive. We could surely find, for example, social psychology journals that have fewer open datasets, or cognitive science journals with more (e.g., Cognition; Hardwicke et al., 2018). Firmly establishing differences between sub-disciplines of psychology would require a separate study.

We recognize that where high open data adoption rates permitted us to locate four datasets quickly, we examined a smaller journal article sample space, and so of course the actual prevalence of open data for that journal may be different. It is also clear that, out of necessity, we selectively sampled journals. Fifteen journals only represent a small number of psychological outlets—Scimago (https://www.scimagojr.com ) identifies 1201 Psychology journals in its 2018 catalog. Nonetheless we predict that many reported outcomes (i.e., historically low adoption rates, wide variability in journal practices) will generalize. Moreover, the present disciplinary differences match our broader perceptions and awareness of the contemporary landscape.

Albeit with a small sample size, we confirmed *post hoc* some broad consistency in journal practice. Journals with higher adoption rates at in the first time period also had a higher adoption rate in the second time period, r(13)=.638, *p* = .011. Yet clearly, journals can and do change open data practices; by policy, and less formally perhaps also by neighbourhood examples (“other authors in this journal share data, maybe I should too”) , incentivization initiatives such as open science badges (Kidwell et al., 2016), and community values (our small sample intimates is that research in social psychology has embraced public data sharing more emphatically than research in applied psychology).

We asked whether the open data prevalence was associated with journal prestige, by using Journal Impact Factor (JIF) ranks from 2017. In other words, we asked whether journals with higher relative impact factors in our corpus publish more papers with open data). We used JIF *ranks* (not JIF values) to mitigate known noisiness and bias (we share many of the widely reported concerns about JIFs, here they merely offered a convenient first-pass score for journals). We found no systematic association with adoption rate at either time window (r(13) = –.401, *p* = .139, and, r(13) = – .279, *p* = .314). Moreover, one journal was a visual outlier, with a much higher adoption rate than others. Removing that case, these non-significant correlations dropped further (r(12) = – .025 & r(12) = .044, respectively).

We came across a revealing issue unique to one journal. Our original search identified seven papers from the same journal (four from the early publication window, three from the later) that explicitly mentioned supplementary material on the journal’s website. However, none of these supplementary files were present. Data likely disappeared during the journal transfer from one publisher to another. This dramatically illustrates the importance of independent repositories and exemplifies issues of dataset preservation already noted in the literature. Our dilemma was this; since we could not access the supplementary materials, we could not identify their contents. Our rules did not clearly define whether we immediately terminated our search (e.g., by reaching four potential datasets) or continued the search since the datasets were not available. We decided to search further, looking for *unambiguous* cases of open data (i.e., those that we could access via an external repository). We then found one unambiguous instance of open data, and we reported an adoption rate based on the total search (in this case, five out of the total 95 empirical papers). This is a very generous adoption rate insofar as we strongly suspected that the supplementary material did not always contain raw data as opposed to summary tables, etc. Obviously, a different process-rule would affect adoption rates for this journal. Our accompanying data deposit describes alternative cell value from different rule choices, but note these did not affect, for example, the significance of impact factor associations above.

#### Take-away message

Provision of public data sharing varies considerably across psychology, but it has been generally been very uncommon, perhaps more so in some areas than others. Moreover, the way in which datasets are described and maintained can be important for their preservation.

### Analysis of the quality of open datasets

**Fig. 4:**
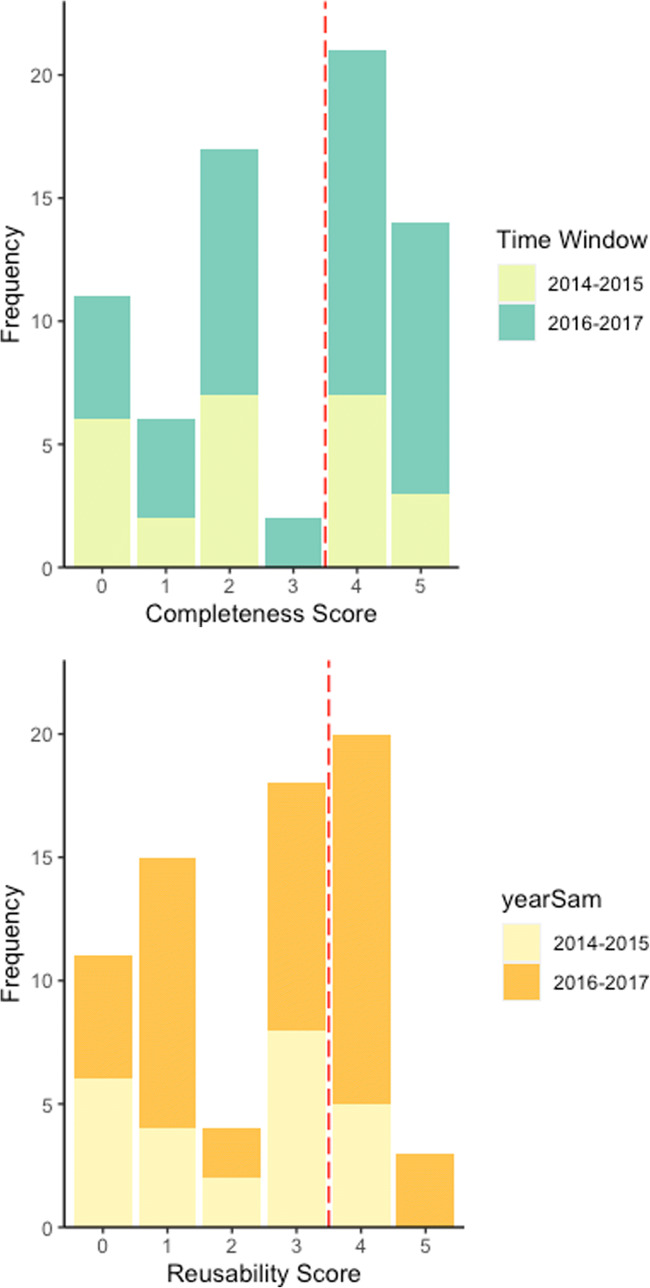
Frequency distribution of dataset functionality scores for (*upper panel*) completeness and (*lower panel*) reusability. A score of 5 indicates exemplary archiving, and a score of 0 indicates no data could be inspected. Studies with completeness scores of 3 or lower (*left of the red dashed line*) are categorised as incomplete / limited-reusability

We acquired 71 datasets over a large search space in psychology and examined the quality of completeness and reusability. Our analysis showed that 51% of these datasets were incomplete, defined by RKLB as having a completeness score of 3 or less. And 68% of datasets were archived in such a way as limit reusability (reusability score of 3 or less). These values are remarkably similar to RKLB analysis (56% and 64%, respectively). It is clear that public data sharing practices in Psychology are variable and often, sub-optimal, just like those in Ecology & Evolution. Figure 4 reports the completeness and reusability scores, formatted similarly to RKLB. By way of comparison, Hardwicke et al. (2018) assessed ‘in-principle reusability’ of psychological data in *Cognition*, through a bespoke assessment of data. They reported that 38% of their datasets failed to meet their quality threshold.

Both completeness and reusability scores were higher in the more recent publication-window (2016/17 compared with 2014/15), but this was not significant (t(58) = .874, and t(58) = .536, both *p*s>.05) and represented a small effect size (completeness: 2.4 vs. 3.1 (*η*^*2*^ = 0.04) and reusability: 2.1 vs. 2.6 (*η*^*2*^ = 0.03)). Bear in mind, however, that as noted in the pre-registration, publication date is a fuzzy variable for determining when researchers embarked on and wrote up their work. Project life span, review times, project write-up times, and publication lags etc. means this is a noisy variable.

RKLB reported that 40% of their non-complete datasets lacked only a small amount of data (i.e., the completeness score was 3). For the psychology corpus, this was only 4%, a value depressed by the presence of missing datasets—self-evidently involved more than just a small amount of data. Nonetheless this suggests that when psychological datasets are not complete, the problems are more severe.

Examining the datasets, we were able to identify, *post hoc*, a feature that can further explain low completeness scores. In particular, five datasets were highlighted as having unexplained data exclusion issues. That is, the dataset comprised *more* participants than were reported in the paper for analysis. Participant exclusion can be an entirely legitimate practice of course—but when it is not possible to determine which participants were excluded, then it is not practically feasible to replicate any findings.

Although some researchers embedded data descriptions within other files (e.g., .xls files or .sav files) it was noticeable that only 14 datasets had a separate ‘readme’ file or data dictionary. This emphasizes that even when researchers are willing to share their data, the extent to which those data can be understood is limited without a simple, independent, and easy to access data dictionary or overview of the deposit.

We asked whether journal status—as before, ranked by JIF—affected data functionality (omitting absent datasets because their quality is not measurable). We found little support for the notion that the research in “flagship” journals offer systematically better-quality open data (r(58) = .152, *p* = .246, and r(58) = .158, *p* = .228).

**Fig. 5:**
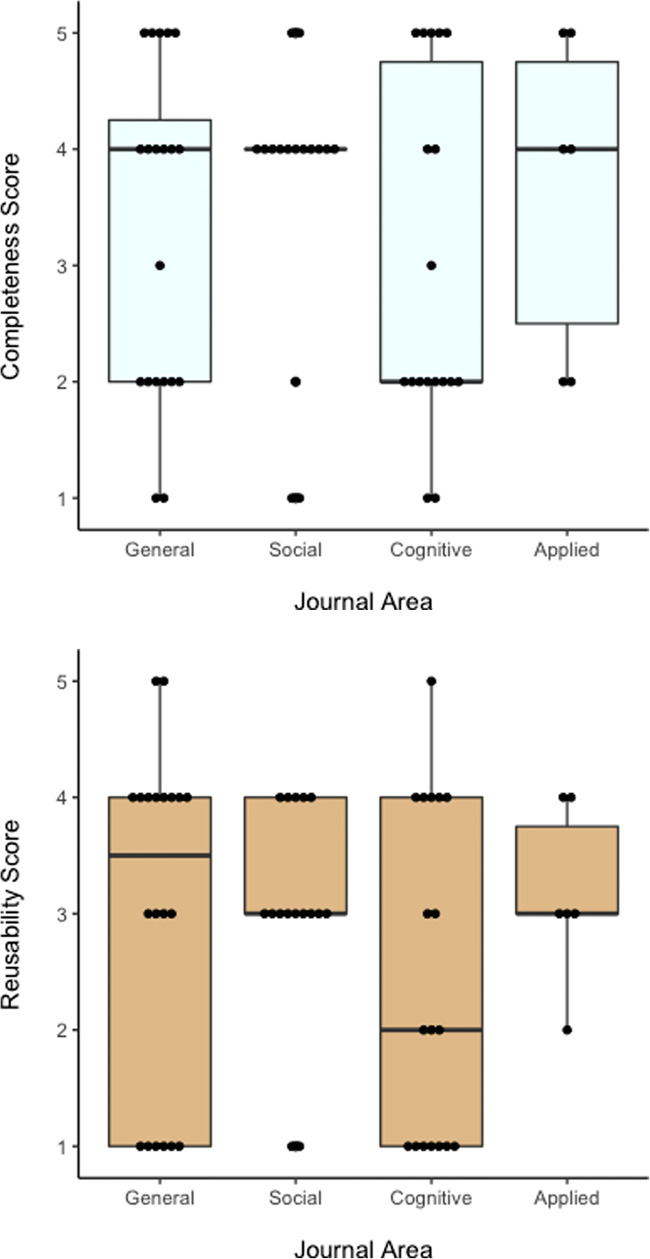
Box-and whisker plots of variability in data completeness (*upper panel*) and reusability (*lower panel*) as a function of journal subfield-categories. Data are based on ratable datasets. *Dots* represents individual data points. The first and third quartile for Social journals are the same value, so no box is drawn here

We report box-plots of data quality across sub-disciplines of Psychology in Fig. 5. These confirm, first, that open data practices are variable wherever they are found, and second, that overall performance was comparable.

Formally, where a dataset is held is neither a component of completeness nor reusability. Nonetheless, RKLB noted that 22% of their corpus involved data archiving through supplementary material—held alongside the article itself—and they laid out longevity risks in this practice (see also, Vines et al., 2014). In the present corpus, 39% of datasets involved supplementary material. This drops to 33% if we exclude data from the one journal with lost supplementary material, a clear evidence-case of course as to why supplementary journal data are problematic. RKLB drew on a single repository source (where data could be outsourced) while our search point was journal articles themselves. We believe this may explain why we found higher use of journal supplementary material. Regardless, all these statistics converge on the conclusion that raw data in science, even when archived, are often fragile, perhaps more so than suggested by RKLB.

Post-hoc we investigated the proportion of datasets lost or at risk of loss—because either they were held as journal supplementary material or linked without persistent identifiers. This amounted to *at least* 46% of the corpus, a calculation overlooking one archive that had a persistent identifier but no data at that address, another that was blocked behind personal permission authentication, and several GitHub links that did not deploy permanent link formats. To detail this issue, our data deposit includes an alphabetised, synthetic (anonymous) version of each dataset location. It is apparent there is an alarming proportion of open datasets in psychology that could be lost or orphaned from source papers, presenting risks for the Findability within FAIR principles.

Imagine that we chose to archive the data for this paper at the following address:

http://www.pc.rhbnc.ac. uk/papers/tr.html

(nb., this address was used by the first author to provide a supplementary text file to an article published in 1998). The fragility of this address is underscored by the way that (a) the institution, then Royal Holloway and Bedford New College (rhbnc.ac.uk) changed its internet address to “rhul.ac.uk” and currently changed again to “royalholloway.ac.uk” (b) the server for the then-psychology department (subdomain “pc”) has been replaced (c) the directory structure for University files has changed so that even setting aside the above issues, the location address would not work. Persistent identifiers are designed to overcome all these issues.

#### Take-away message

As found in other areas of science, the majority of open datasets in Psychology were incomplete and of limited reusability. We found a particular problem with data exclusions. We also describe substantial issues with dataset locations, putting them at risk of loss, or becoming orphaned from source papers, or undergoing non-audited changes.

### Alternative analysis of data quality

Given the framing of this project throughout as a comparison of dataset functionality with Ecology & Evolution, it was critical to replicate the RKLB procedures to judge the data functionality. The measures are not without limitations, however. For example, the presence of meta-data or a codebook contributes both to the completeness and reusability score. Whilst meta-data are pertinent to each quality dimension, this inevitably restricts their independence. Indeed, the association between completeness and reusability was high, r(58) = .775, *p* < .001, as RKLB reported also.

**Fig. 6:**
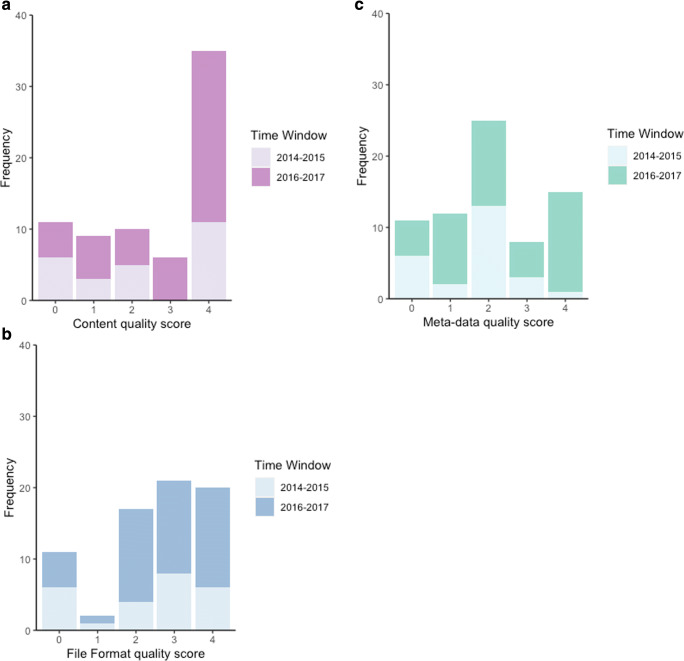
Distribution of dataset quality scores (0–4 for increasing quality) focusing on **a** content (completeness); **b** File format; **c** Meta-data

Accordingly, we developed post-hoc a complementary set of three data-quality measurements that were more independent of each other, focusing on data completeness, file format, and metadata. In each case, a dataset was given a score as follows; 4 (exemplary); 3 (minor issue); 2 (major issue); 1 (not interpretable); 0 (no dataset to evaluate)—see data deposit for more details. This deliberately provided a coarse-grain differentiation between datasets. These additional scores also reinforce how the present analyses are not just reliant on the RKLB scales. 42% of datasets had at least major issues with completeness, 42% had at least major issues with file format, and 68% had at least major issues with meta-data (see Fig. 6). These figures support the quality profiles already reported, but emphasizes that metadata—the description and explanation of the data—is the most problematic dimension of the dataset corpus.

Unsurprisingly, we found a strong correlation between a combined completeness and reusability score as one variable and a combined score from the alternative three measures as another, r(58) = .807, *p* < .001. However, while content quality correlated with metadata, r(58) = .555, *p* < .001, it was de-coupled from file format, r(58) = – .048, *p* = .716, and metadata only weakly associated with file format, r(58) = – .262, *p* = .043. Complete, well-labelled datasets (i.e., high-quality content) can be found across file types, even files types which are not necessarily accessible. We conclude that data quality is indeed a multi-dimensional construct with several separate components coming together to provide the most useful open data deposits.

We have attempted to shield the sources of our datasets (i.e., the original empirical papers) because convergent with RKLB, our goal is to profile aggregate practices, not to applaud or criticize specific authors or groups. It is essential to keep in mind that although many open datasets are sub-optimal in terms of completeness and reusability these authors have nonetheless attempted to share data. That is, for whatever reason over 95% of papers we initially searched *did* ***not*** *contain identifiable open data*. These data are not just incomplete, they are non-existent. These data are not reusable, they are completely inaccessible. Evaluations of open data quality profiles need to be contextualized with open data prevalence, even in recent publication periods when the benefits and importance of open data practices have been clearly identified (Castro, Hastings, Stevens & Weichselgartne, 2015; Munafo et al., 2017).

#### Take-away message

Provision of meta-data (data descriptions and contextual information) is a particular weak point of psychological datasets, and can render the data difficult or impossible to interpret. File formats that may be difficult or impossible for others to access also reduce the functionality of many datasets.

## General discussion

If psychological research is going to become truly open, then we need to recognize that public data sharing is important. But on its own, it is not enough. Rather we must strive for *high-quality, effective open data*. Sub-optimal open data, through for example carelessness, lack of foresight, or lack of relevant experience and training, can substantially impede data use. Given that the majority of our psychological datasets were neither complete nor re-usable (as defined by RKLB), we encourage a step-change in recognizing not just that open data should become more common, but simultaneously that open data becomes more functional and optimized.

We found that psychological datasets show a very similar quality profile to those sampled by RKLB. In both cases, the majority of datasets were incomplete, and almost two-thirds had limited reusability. Whilst we were unable to acquire sufficient open datasets at exactly the same time period as RKLB (2012/13) it is clear that problematic practices have persisted through to at least 2016/17 within psychological science. However, such issues are not specific to Ecology & Evolution, nor to Psychology. Rather, data point to the *generality* of open science practices and opportunities, over and above disciplinary phenomena, a conclusion that is supported by convergent conclusions across social science (Hardwicke et al., 2020). The implications of this shouldn’t be underestimated, since “crises” or problems are often cast in terms of the fields in which they are examined, even though reproducibility is a concern for most, if not all science (Baker, 2016).

RKLB briefly, commented on one disappointing feature of their data corpus, finding “poorly identified data unrelated to the paper”. Our analyses show that this limiter is found in psychological datasets also. That is, we too found cases of missing data. We also recorded another prevalent issue with data completeness more specific to Psychology, additional data. In some cases, there were *more* participants contributing to the dataset than the reported analysis. It is common in psychology to exclude participants for legitimate reasons and any additional data is not problematic when the paper or readme clearly defined which participants were excluded from the analysis. However, on some occasions, the paper reported participant exclusions but it was not possible to identify which participants in the dataset were excluded from the analyses. Furthermore, some exclusions were not reported in the paper, but we presume must have taken place.

### Limitations

First, shared data are not always easy to find, html versions of paper and pdf versions of papers sometimes made the links differentially salient, we may have not found all of the open data available. It was very much in our interests to find datasets where possible, since our protocol dictated self-terminating search and absence of source data meant looking through additional papers. If we missed any datasets, so could others, including scientists looking to re-use them. It is also noteworthy that open data prevalence rates here converge with those coincidentally reported for the same time period across social science by Hardwicke et al. (2020).

Second, we did not assess corpus datasets that existed independent of the research papers (e.g., census data). Our focus rather was on novel data specific to the research papers being published. One might easily imagine that with large datasets existing across many papers or independent of papers, that completeness and usability would be high, since they would be designed with these constraints in mind.

Third, we focused on one protocol for inspecting and scoring dataset quality—based on RKLB. This was fundamental to the objective of creating commensurate data for psychology. However, this does not imply that their methodology is the only way to evaluate datasets. Indeed, we also investigated more focused assessments of dataset profiles. Importantly, these, along with overlapping but bespoke approaches taken in other recent work (Hardwicke et al., 2018), all converge in pointing to the scale of the dataset functionality problem and the heterogeneity in quality. Notably, not only did Hardwicke et al. (2018) derive estimates of *in-principle reusability*, they also looked at a subset of datasets and measured *analytic-reusability* of the data—that is they attempted to reanalyse the data and reproduce statistical outcomes from the target papers. This procedure identified many further reusability issues with the datasets. The conclusion relevant here is this—the metrics we describe for completeness and reusability are best-case estimates. For all the reasons detailed in this paper, and the evidence from Hardwicke et al. (2018), we expect that our scores over-estimate the ability to exactly replicate analytic outcomes.

Fourth, we sampled datasets from throughout the publication windows 2014–2017 (so our corpus corresponds closely to those from Hardwick et al. (2018; 2020), where sampling was Jan 2014–April 2017 and March 2014–March 2017, respectively). We have shown how data functionality is highly similar to that found in Ecology & Evolution in 2012/2013–demonstrating generalizability across science and across time. Additionally, there is only a small effect size in our analysis for changes over time in data quality. Whilst it is *possible* that dataset quality has somehow changed dramatically since, our analysis makes us confident in predicting that until there is wider recognition of the current problem, and the opportunities for solutions, many practices will continue to change slowly. For example, more widespread use of data repositories with easy-to-create DOIs (such as OSF) may improve some facets of the situation (data held by journals as supplementary files may correspondingly disappear). Yet, until the emphasis shifts, from increasing open data provision towards a broader appreciation of also changing open data quality, we do not anticipate step changes in the profile we describe.

### Seven recommendations (and their purpose)

- **Use third-party repositories (to help maintain data Findability as part of FAIR).** We emphasise the argument from RKLB that open data should be available through independent repositories where appropriate access and maintenance provisions can be established while journal supplementary data should be avoided. The repository should provide a persistent link such as a DOI (easily available through OSF but many other options exist). This would help counter hyperlink rot, and the inadvertent loss of data access through website changes. We demonstrated that a large proportion of sampled datasets are already unavailable or at risk of loss. Where feasible, we also suggest that journals check that DOIs are functional and point to the correct address for open data. Open data will then be made much more resilient for longer-term access.
- **Fully describe the dataset (to improve its functionality and interoperability).** As we have demonstrated, data completeness and data reusability are problematic for many shared datasets. The provision of high-quality metadata is important to each dimension, and notably it is one of the weakest aspects of the datasets in our corpus. Authors appear to focus on the numbers (for quantitative data) at the expense of their meaning and context. Numeric data are nearly always difficult to understand without guidance about their provenance, their context, and their details.
- **Journals could provide clear, practical open data guidelines (to improve data quality, especially interoperability and reusability).** Authors should be provided with clear and transparent guidance about the expectations for functional data provision, that address completeness of data, file format and meta-data. Where feasible, advice should indicate how to provide all the available raw data (not just those which are reported). Exemplars of well-organized, functional datasets would likely help. Data standards are not static, nor are they uniform across psychology. However, since authors cannot anticipate all current or future opportunities for dataset use, journals could facilitate the promotion of current dataset best practices (for an example, see UKRN data sharing primer). This would address concerns from researchers about the lack of training in how to optimize public data sharing (Houtkoop et al., 2018).
- **Authors should ensure a long-term, accessible version of their data (to improve reusability).** There may be good reasons for authors to include data in proprietary formats, because of the functions or processes that can be captured that way. Yet authors can usually *also* include a standard, plain-text version of the data to ensure users are not locked out by commercial, restricted or obsolete software. It may be helpful for authors to provide a clean, as-analysed dataset whilst *also* providing the raw data that were used to derive these values.
- **Provide clarity about the authoritative version of data (to ensure credibility of data and its reusability).** As part of the process of ensuring data have persistent identifiers and long-term access, we recommend that authors carefully configure the archive to confirm they provide non-editable copies of files or transparent version-control. This is to ensure that once archived, data remain a stable version-of-record in the same way that is expected of a research publication. Dataset users need to have confidence in the integrity of the data as a stable entity, which current practices do not enforce.
- **Remember that there are ways to share sensitive data (overcome obstacles to sharing data)**. The phrase “as open as possible, as closed as necessary” is a useful guiding principle (Landi et al., 2020). Even in cases where it is not feasible to provide all raw data perhaps due to ethical, legal or other reasons (see discussion in Ross, Iguchi & Panicker, 2018), some data is very likely to be better than none at all. This can be argued as especially relevant for applied research—such research may drive policy and in our analyses applied psychology journals had particularly poor adoption rates. Appropriate restriction on *some* data should not be taken as reason to withhold *everything* (for a discussion on the changing nature of hyperconnected data, see Dennis, Garrett, Yim et al., 2019). Recent proposals for generating synthetic datasets may help to address this (Quintana, 2019). Synthetic datasets mimic original datasets by retaining their statistical properties and relationships between variables, but no record in the synthetic dataset represents a real individual. As an example here, our file of partially scrambled web addresses provides a simple synthetic dataset as it allows other researchers to replicate high level results (e.g., the number of available datasets and online repositories utilised) without revealing information about the original publication or associated journal. Moreover, for some experimental designs, aggregated or processed data sharing such as variance-covariance matrices may permit some meaningful follow-up analysis to be attempted.
- **Standardize how open data is identified at a journal level (signposting the invitation to provide data and emphasise findability).** At a journal level, we recommend that published articles provide a standard route to the identification of datasets and other material. If authors know exactly where in their article to describe their data management plans, this would provide a tangible structural incentive and behavioural nudge for authors to provide open data where feasible. If readers know where to look, data use will be much simplified. It would also help automation of dataset identification. Note that journal requirements for data availability statements may not produce compliance in all cases (Federer et al., 2018). Standardizing how open data is identified should increase prevalence of open data.

**We argue that the provision of open datasets is a valuable, important exercise that should be the norm rather than the exception**. Obstacles to accessing data and analysis syntax have existed for some time (Wicherts, Borsboom, Kats & Molenaar, 2006; Wicherts & Crompvoets, 2017) but many solutions exist and authors can offer high-quality deposits. Open data is a manageable albeit time consuming target, especially where thoughtful and careful curation takes place and issues of anonymity must be managed. The field should recognize the value, and the temporal and cognitive costs, whilst promoting the potential reward and benefits to Psychology. As Mons et al. (2017) note, “it is very burdensome to peer review the quality of data at the time they are first published” and therefore ways to balance the importance of open data alongside author and journal overheads are important.

In developing the recommendations above, we have avoided one obvious potential suggestion: to make open data compulsory. Wicherts & Crompvoets (2017) articulate just this argument for analytic code provision. However, bear in mind that RKLB analysed data from journals with strong data deposit requirements—clearly it not a necessary and sufficient catalyst on its own for high-quality data (see also Federer et al., 2018). Consequently, we have focused here on ways to engage with and encourage the curation of useful data.

## Conclusions

Positive change has and does continue to occur in frequency of open data provision. Yet when public data sharing happens it often exhibits problems with completeness and reusability, similar to findings in other disciplines. We have therefore provided a series of straightforward recommendations that can help promote further change. These include specific and simple steps for both journals and individuals which together with appropriate training will improve the functionality of open data.

## Supplementary Information

ESM 1 (DOCX 963 kb)

## Footnotes

1 In this paper, reported analyses by default were planned or pre-registered. We use the term *post-hoc* as an explicit marker that analyses had not been pre-registered. We do not use the term exploratory because some of our pre-registered questions are descriptive, not inferential, and this term could cause confusion.
