# Supplementary material for: Opening Pandora’s Box: Peeking inside Psychology’s data sharing practices, and seven recommendations for change
Source: Behav Res Methods. 2020 Nov 11;53(4):1455–68. doi: 10.3758/s13428-020-01486-1 (PMC8367918; doi:10.3758/s13428-020-01486-1)
Supplement: Supplementary file 1 — (DOCX 963 kb) [file 13428_2020_1486_MOESM1_ESM.docx]

**Supplementary Materials**

For pre-registrations, data, annotations and plot codes, see: <https://osf.io/2fpgc>. For comparison data made available by RKLB, see: <http://dx.doi.org/10.6084/m9.figshare.1393269>

The assessment protocol for data functionality was described in tabular form by RKLB and is reproduced below:

**
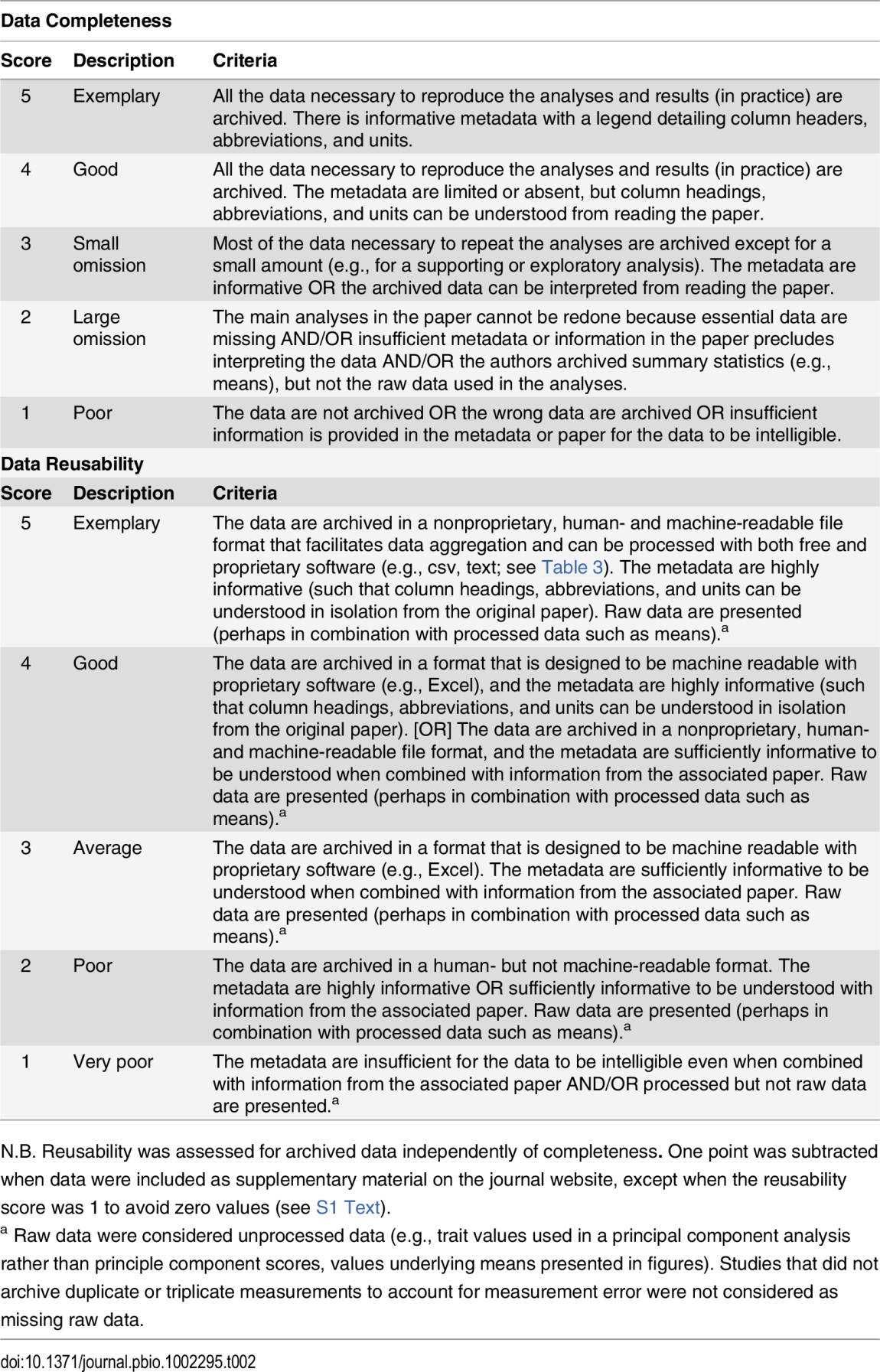
**
